# Suicide mortality rates in Japan before and beyond the COVID‐19 pandemic era

**DOI:** 10.1002/pcn5.188

**Published:** 2024-04-01

**Authors:** Motohiro Okada, Ryusuke Matsumoto, Eishi Motomura

**Affiliations:** ^1^ Department of Neuropsychiatry, Division of Neuroscience, Graduate School of Medicine Mie University Tsu Japan

**Keywords:** COVID‐19, Japan, suicide

## Abstract

Statistical analyses from Japan reported increasing suicides in 2020, first in the world, proving the severity of the public health crisis during the COVID‐19 pandemic; however, so far, international suicides have not been shown to be objectively increasing at population level. Followed studies reported the existence of a substantial heterogeneity of suicides among subgroups and time‐lag impacts. Against public health crisis in Japan, policymakers, psychiatrists and public health personnel should prioritize improving suicide prevention programs following evidence‐based policymaking. Understanding how/what factors relate to the COVID‐19 pandemic and what other factors have shaped the increasing suicide numbers since 2020 through objectively well‐controlled/fine‐grained analyses of high‐quality longitudinal/cross‐sectional data at the individual, regional, and national levels is important for identifying the reasons for the recent trend. For this purpose, this study examined suicide statistics, statistical analysis methods, and their interpretations. Recent analyses suggest an increased suicide risk among females <50 years and males <30 years in 2020–2022. Notably, time‐series analyses revealed that adolescent suicides began increasing before the pandemic, while working‐age female suicides sharply increased synchronously with the pandemic outbreak. Causality analyses suggest that social issues facing Japan and recent global psychosocial and socioeconomic transformations are risk factors for suicide in high‐risk groups. Finally, this report demonstrates the importance of providing appropriate support based on an objective understanding of individuals who are at risk for suicide, without being bound by traditional established knowledges.

## Graphical abstract

Deceasing trends of age‐standardized suicide death rates (SDRs) were attenuated in late in 2010s. SDRs in Japan increased from 2020 compared with predicted SDRs detected by both autoregressive integrated moving average and interrupted time‐series analyses models.

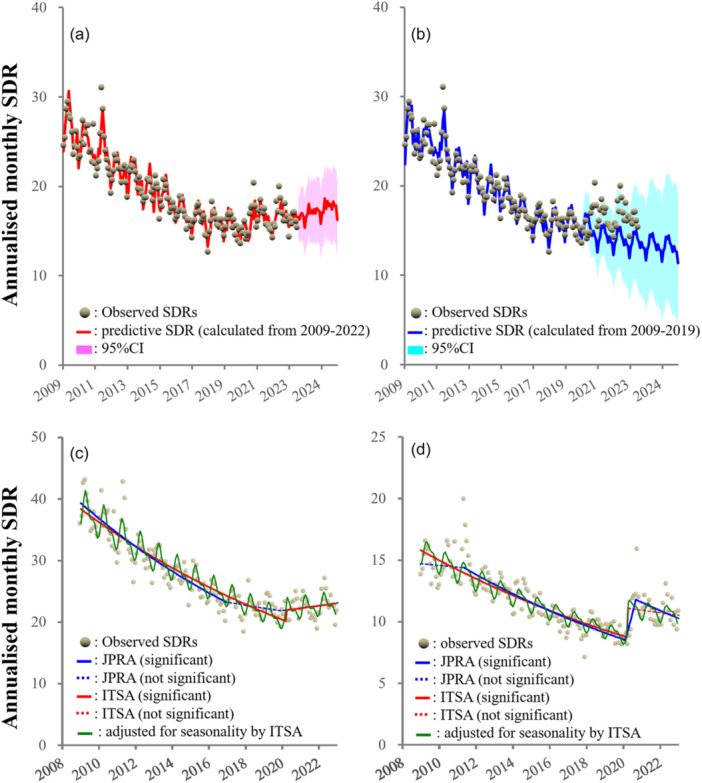

## INTRODUCTION

Crude suicide mortality rates per 100,000 population (CSMRs) in Japan have varied widely over the past quarter‐century. Until the early 1990s, the CSMR of Japanese males was ~17–18, which was not higher than other Organisation for Economic Cooperation and Development (OECD) countries.^1^ However, following the collapse of the asset bubble in 1991 and the 1997 Asian economic crisis, annual suicides in Japan increased drastically in 1998 (1997, 24,3921; 1998, 32,863). This increase continued until 2019 (maximum 34,427 in 2003).^2^, ^3^, ^4^ To address this crisis, the Japanese government enacted the Basic Act on Suicide Prevention in 2006 and the General Principles of Suicide Prevention Policy (GPSPP) in 2007 to provide financial support for prefectures/municipalities to implement comprehensive regional suicide prevention programs.^5^ According to these policies, in 2009, the Japanese government began to contribute funds to prefectures/municipalities via the Emergency Fund to Enhance Community‐Based Suicide Countermeasure (EFECBSC) of the Ministry of Health, Labour and Welfare (MHLW) to enhance comprehensive regional suicide prevention programs.^5^, ^6^, ^7^, ^8^, ^9^, ^10^ The persistently high suicide mortality rates, which lasted >10 years, have consistently decreased since 2009–2019.^7^, ^8^, ^9^, ^10^

Annual suicides increased from 20,169 in 2019 to 21,081 in 2020, sustained until at least 2022.^11^, ^12^, ^13^, ^14^ Given the timing of the COVID‐19 pandemic, most reports have concluded that the increase was impacted by psychosocial and/or socioeconomic deterioration associated with the pandemic.^15^, ^16^, ^17^, ^18^, ^19^, ^20^, ^21^, ^22^, ^23^, ^24^, ^25^, ^26^ However, on closer scrutiny of these reports, it was demonstrated that, with the exception of a few reports,^12^, ^13^, ^14^, ^27^, ^28^ most findings regarding the CSMR or age‐standardized suicide death rate (SDR) were inferred based on the increase in comparison to before the pandemic or synchronized with the pandemic, without analyzing the underlying causes, therefore the reasons for the increased SDR/CSMR in 2020–2022 remain to be clarified.

On May 5, 2023, the WHO declared “COVID‐19 no longer represents a global health emergency”, ending a >3‐year pandemic.^29^ Going forward, continuous fine‐grained observation of fluctuations of suicides is essential for public health because many hope for rapid improvement of increasing suicide in the postpandemic era. However, some studies have reported that suicides in some subgroups began increasing prior to the pandemic, which cannot be ignored,^12^, ^13^, ^14^, ^25^, ^26^, ^28^ therefore complicated interactions among various factors may underlie the increase in suicides in Japan since 2020. Accordingly, evidence‐based policymaking concept, logic and critical thinking,^30^ and the identification of the underlying causes of decreasing CSMR/SDR during 2009–2019 and increasing from 2020 to 2022 could contribute to the improvement of suicide prevention program planning. Indeed, the MHLW provided financial support for the Suicide Prevention Measures Project in response to COVID‐19 in 2020 and 2021,^4^, ^31^, ^32^ but it did not suppress increasing CSMR/SDR. We must therefore reconsider whether the increase after 2020 is due to the pandemic or other factors, and suicide prevention programs should be revised accordingly.

## BASIS OF STATISTICAL MEASURES FOR SUICIDE MORTALITY TRENDS

### Importance of fluctuations in population/age‐distribution

It is generally not incorrect to compare the annual/monthly suicides number in a region with the previous year, as long as the demographic composition (age‐distribution/population) within the region does not change significantly. However, while suicide numbers in Japan decreased between 2020 and 2021 (21,081 to 21,007),^11^ the CSMR increased from 16.58 to 16.59 due to the national population decreasing from 127,138,033 to 126,654,244.^33^ This discrepancy indicates the importance of population‐based standardization of suicide mortality.

When the prevalence risk is independent of age, the regional age distribution is considered unimportant when comparing suicide statistics among regions. However, CSMRs disaggregated by age in Japan have increased in an age‐dependent manner in the <60 years population.^7^, ^8^, ^12^, ^28^, ^34^, ^35^ Therefore, when suicide risk is age‐dependent and the age distribution in regions changes over time, age distribution becomes an important demographic variable for suicide statistics. In Japan, which has a decreasing birth rate and aging population (predominant in rural areas), even if CSMRs for each generation remain stable, national‐level CSMRs may increase due to aging. For example, the Mie and Iwate prefectures are well known to have the lowest and highest CSMRs in Japan, respectively. In 2010, the female CSMRs in Mie and Iwate prefectures were the lowest (10.45) and the highest (22.17), respectively, whereas in 2018 they were 13.01 and 13.86, respectively (the second highest and highest CSMRs, respectively). Conversely, the female SDRs of Mie and Iwate (based on population age distribution in 2009) were 12.78 and 12.18, respectively, with the female SDR of Mie prefecture being the highest in Japan, therefore suicide mortality standardized by age distribution is an important statistic. Most studies of international trends in suicide mortality provide standardized death rates using the WHO World Standard Population (WSP) model^36^ or the European Standard Population (ESP) model.^37^

**Figure 1:**
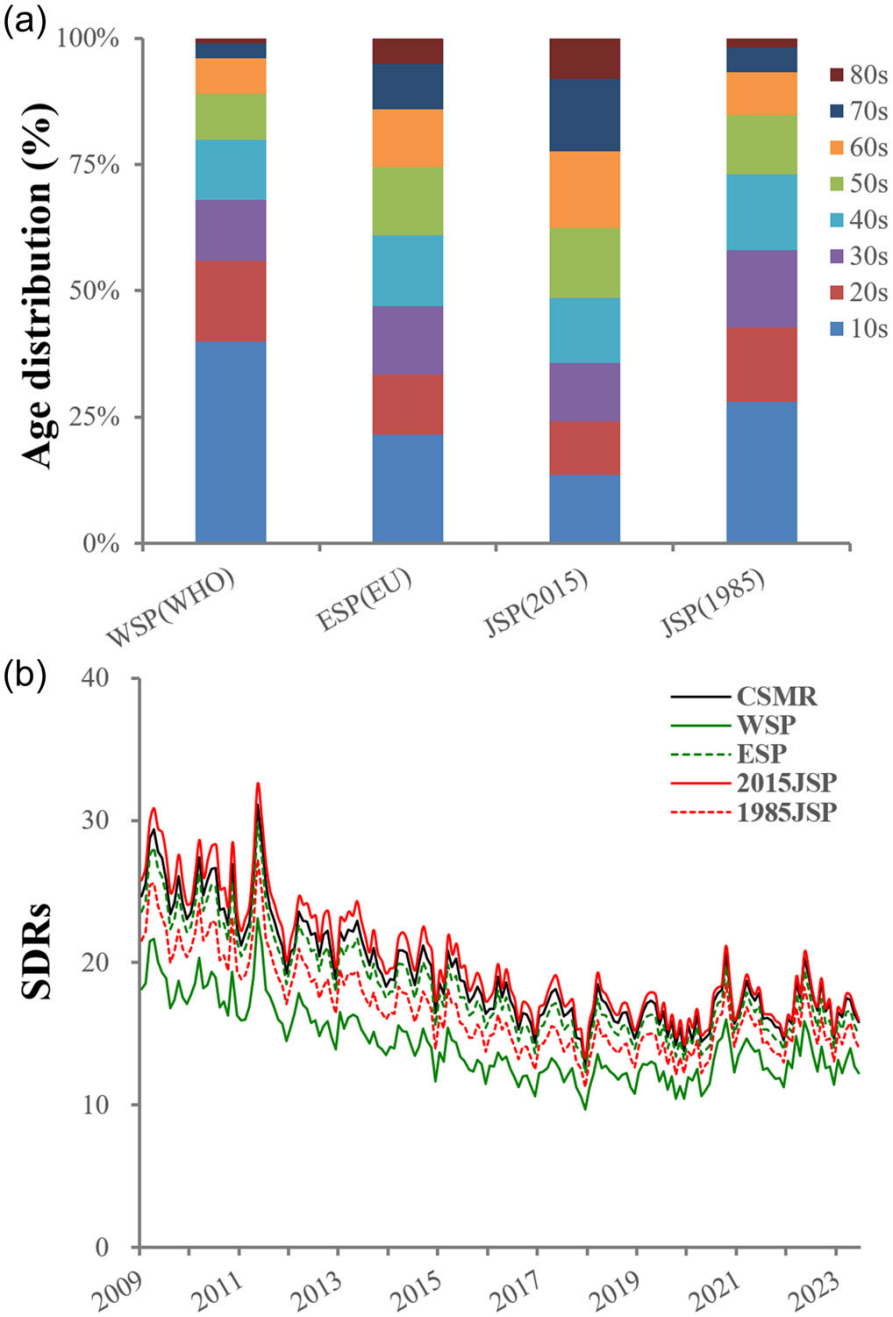
Comparison of age distribution among international standard population models and age‐standardized suicide death rates per 100,000 in Japan from January 2009 to June 2023 calculated using international standard population models, respectively. 1985JSP, Japanese standard population model based on the population in 1985; 2015JSP, Japanese standard population model based on the population in 2015; CSMR, crude standardized suicide mortality rate per 100,000; ESP, European standard population model; SDRs, suicide death rates; WSP, WHO world standard population model.

Traditionally, the standardized mortality ratio in Japan has been calculated using the Japanese standard population model, which was based on the population from 1985 (1985JSP). However, the population of Japan is aging rapidly. Accordingly, in 2022, the model was revised based on the population distribution from 2015 (2015JSP).^38^ From now, SDRs will be calculated using the 2015JSP model. Therefore, the SDR in the present study was calculated using the 2015JSP model (Figure 1).

### Analyses of temporal fluctuation in suicide mortality

The most fundamental issue in suicide statistics is the inability to establish reliable control data, such as clinical trials.^39^, ^40^, ^41^ Therefore, identifying excess suicide mortality via statistical modeling is considered difficult, since suicide is a phenomenon that results from complicated interactions among various factors.^41^, ^42^, ^43^, ^44^, ^45^, ^46^ To overcome these issues, recent studies have analyzed the temporal fluctuation or excess mortality of SDR/CSMR using four main models: (1) comparison between previous averages and observed data using an analysis of variance (ANOVA) or linear mixed‐effect model (LMM),^23^, ^28^, ^47^ (2) comparison between predicted values calculated from the seasonal autoregressive integrated moving average (sARIMA) and observed value using ANOVA/LMM,^24^, ^27^ (3) detection of impacts of intervention (changing trends and discontinuities) using interrupted time‐series analysis (ITSA),^13^, ^20^, ^25^, ^47^ and (4) detection of fluctuations of trends using joinpoint regression analysis (JPRA).^12^, ^13^, ^26^, ^28^

#### Comparison with average before the pandemic

To determine the excess mortality in the target period (year), when the data were stable, comparison to the average of the preceding 5 years, not just the previous year, is generally recommended.^48^, ^49^, ^50^ As a typical example, the Office for National Statistics in the UK publishes the average deaths of the previous 5 years as the average of past “normal” deaths.^49^ However, in the 5 years prior to the pandemic (2015–2019), Japanese suicide numbers continuously decreased by approximately 20% (from 24,025 in 2015 to 20,169 in 2019).^4^, ^11^ It is necessary to carefully consider whether this 20% decrease is stable. In fact, when SDR/CSMR after the pandemic outbreak was compared with the average SDR/CSMR for 3 years (2017–2019) and 5 years (2015–2019) previously, the statistical analyzing results were quite different.^23^, ^28^ Because the SDR/CSMR disaggregated by factors during the pandemic was found to be significantly higher than the average from 2015 to 2019, it can be concluded that suicides increased after the outbreak in that subgroup. However, the lack of a statistically significant increase does not mean that there was no increase in the risk of suicide. The increase during the pandemic may have been underestimated due to the strong decreasing trend in the prepandemic period.^28^

#### Seasonal autoregressive integrated moving average model

**Figure 2:**
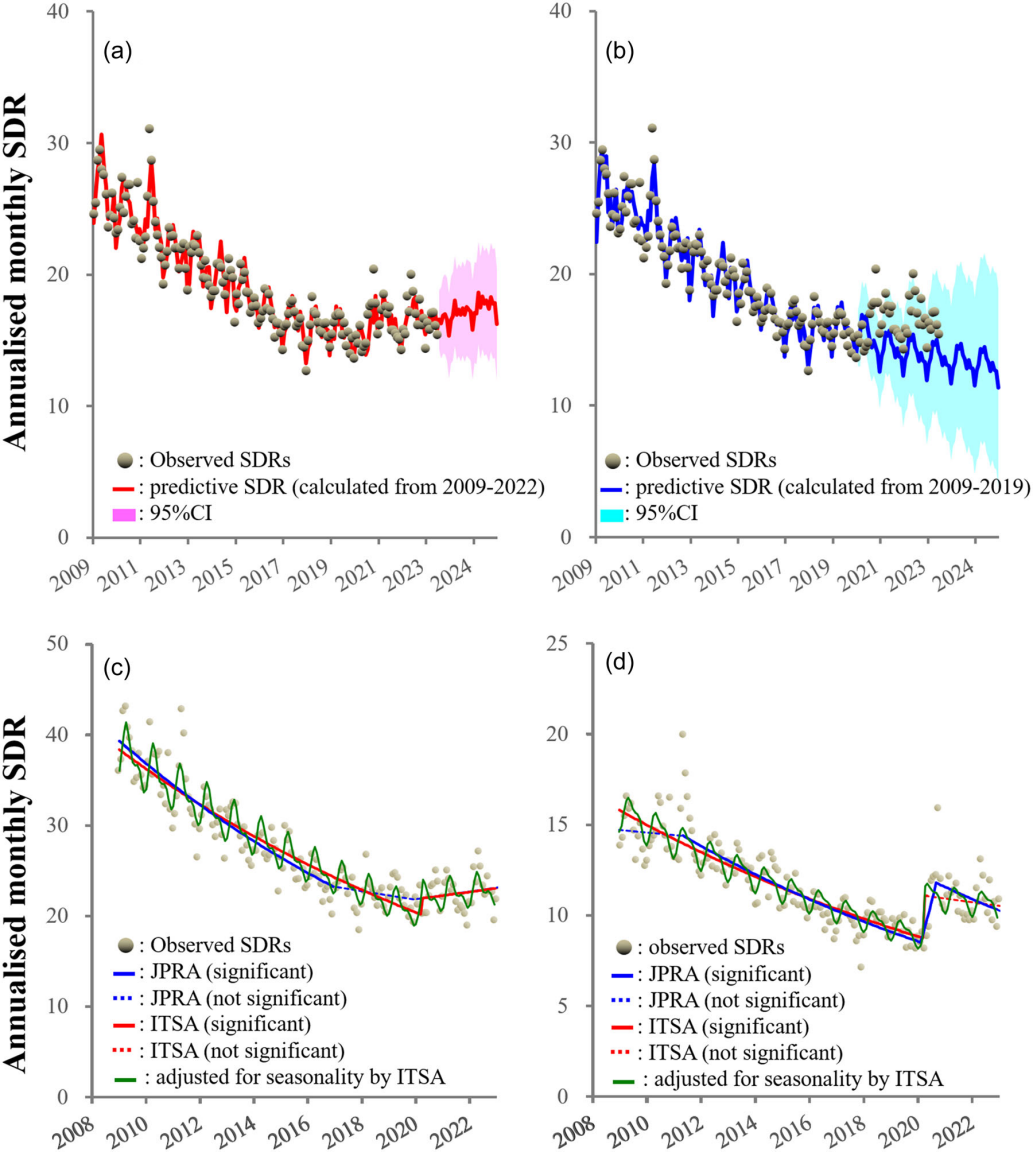
Fluctuations of annualized monthly SDRs in Japan. Predicted SDRs calculated by sARIMA using observed monthly SDRs (a) from 2009 to 2022 and (b) from 2009 to 2019. The red and blue regions on the right of each graph indicate the 95% confidence intervals. Fluctuations of the SDRs of (c) males and (d) females calculated by ITSA and JPRA from January 2009 to June 2023. Blue and red lines indicate the results calculated by JPRA and ITSA, respectively. Solid and dotted lines indicate the significant and not significant trends of SDR detected by statistical analyses (JPRA and ITSA), respectively. Green lines indicate the adjustment for seasonality by ITSA. In all panels, gray circles indicate the observed annualized monthly SDR values. ITSA, interrupted time‐series analysis; JPRA, joinpoint regression analysis; sARIMA, seasonal autoregressive integrated moving average; SDR, suicide death rate.

The sARIMA is an established statistical method that is commonly used for national‐level economic forecasting in various OECD countries, including Japan.^1^, ^51^ We have already reported the comparison of suicides between predicted values calculated by the sARIMA and observed SDR/CSMR until 2021 using panel data.^23^, ^24^, ^27^ In particular, sARIMA exhibits strong advantages in future forecasting, including trends and seasonal variability.^52^ Indeed, the combination analysis of the sARIMA with multivariate analysis of variance/LMM detected turbulence of seasonal fluctuations in the female SDR/SMR, a transient decrease in the female SDR/CSMR at the pandemic outbreak, and a gentle upward shift in the SDR/CSMR of some groups of males and females.^23^, ^24^, ^27^ However, when there is markedly significant irregular variability in trends or seasonal variability in the observed period, the accuracy of the forecast is reduced.^52^ Considering that the pandemic lasted for 3 years, there are some issues to consider when implementing the sARIMA for the predicted values in the target period. Due to the statistical characteristics of the ARIMA (predicting values based on previously observed data), the longer the prediction period is, the greater the error in the predicted values. When the SDR for 2024–2025 was predicted using the sARIMA based on values from 2009 to 2019 and 2009 to 2022, the results were quite different (Figure 2). The predicted SDR for 2024–2025 based on 2009–2019 values shows decreasing trends with large deviations, while the SDR predicted from 2009 to 2022 values shows increasing trends with relatively small deviations (Figure 2).

#### Time‐series analyses

ITSA is an effective method for analyzing the impacts of interventions on trends and discontinuities (i.e., upward/downward shifts at the intervention period).^53^, ^54^, ^55^ ITSA has the advantage of being able to incorporate various options (e.g., parametric and nonparametric regressions, seasonal variation, and panel data analyses).^53^, ^54^, ^55^ Based on these advantages, a number of studies have used ITSA to analyze the impacts of the pandemic on SDR/CSMR fluctuations.^13^, ^20^, ^25^, ^47^, ^56^ However, ITSA is unable to detect any fluctuations that are unknown during the observation period. Thus, if a decreasing trend in suicide mortality has already changed before an intervention, ITSA may overestimate an increasing trend or positive discontinuity in fluctuations after the intervention.^13^

JPRA (also known as the segmented regression model) fits the simplest joinpoint model that the trend data allow and identifies significant points where trends change.^57^ JPRA is used to analyze the number of connection points by calculating the sum of residual error squares between the fitted value and the true value. The method uses model fitting to divide a long‐term trend line into several trend sections.^12^, ^26^, ^28^ A comprehensive review of the statistics and underlying methodology applied in JPRA has been reported.^57^ The connecting points of different trend segments are called joinpoints. Thus, JPRA is a powerful statistical method for detecting unknown joinpoints (transformed trends and discontinuities).^57^ Compared with ITSA, JPRA can flexibly detect joinpoints in suicide mortality over observation periods but cannot directly detect the impacts of the target intervention. Which statistical methods are appropriate for detecting the characteristics of temporal fluctuations in suicide in Japan from 2009 to 2023 is an important point for discussion, since suicide results from complicated interactions among various factors.^39^, ^40^, ^41^, ^42^

The fluctuations in SDR from January‐2009 to June‐2023 detected using ITSA and JPRA indicated different temporal patterns (Figure 2). In males, ITSA detected decreasing trends in the male SDR until the pandemic (2009–2019), but there was positive discontinuity (sharply increasing) synchronized with the pandemic outbreak followed by an increase during the pandemic. However, JPRA detected two joinpoints in 2017 (decreasing to unchanging) and 2020 (unchanging to increasing) without positive discontinuity. Interpreting the nonidentical temporal fluctuations of the male SDR detected by ITSA and JPRA, based on the statistical characteristics of ITSA and JPRA, male suicides showed a consistent decrease until 2016, which was attenuated from 2017 to 2019. ITSA probably overestimates the transformation from decreasing to increasing trends during the pandemic as a positive discontinuity due to the underestimation of attenuated decreasing trends before the pandemic (from 2017 to 2019). In females, ITSA also detected a consistent decrease in the female SDR before the pandemic and a larger positive discontinuity during the pandemic outbreak followed by no change during the pandemic. The effect size of positive discontinuity synchronized with the pandemic of the female SDR seemed to be larger than that of the male SDR. JPRA detected three joinpoints in 2011 (unchanging to decreasing), 2020 (decreasing to increasing), and 2021 (increasing to decreasing). ITSA may overestimate the sharply increasing trends after the pandemic as positive discontinuity (within 1 month increase during the pandemic), resulting in a gradual but significant decrease since 2021, which may be underestimated as unchanging. These findings indicate that JPRA may be suitable for analyzing the temporal fluctuations of SDR/CSMR in Japan since 2009.

### Exploring high‐risk groups for suicide during the pandemic

The group at risk for suicide during the pandemic is almost the same as the group that showed an increased SDR/CSMR during the pandemic. When planning revised suicide prevention programs, it is important to clarify whether the increased SDRs/CSMRs of high‐risk groups during the pandemic are due to pandemic‐associated factors or other factors that occurred before or after the pandemic. Time‐series analyses can provide essential information for identifying high‐risk groups by clarifying the bases for the temporal relationships between increasing SDRs/CSMRs disaggregated by different factors and the pandemic.

**Figure 3:**
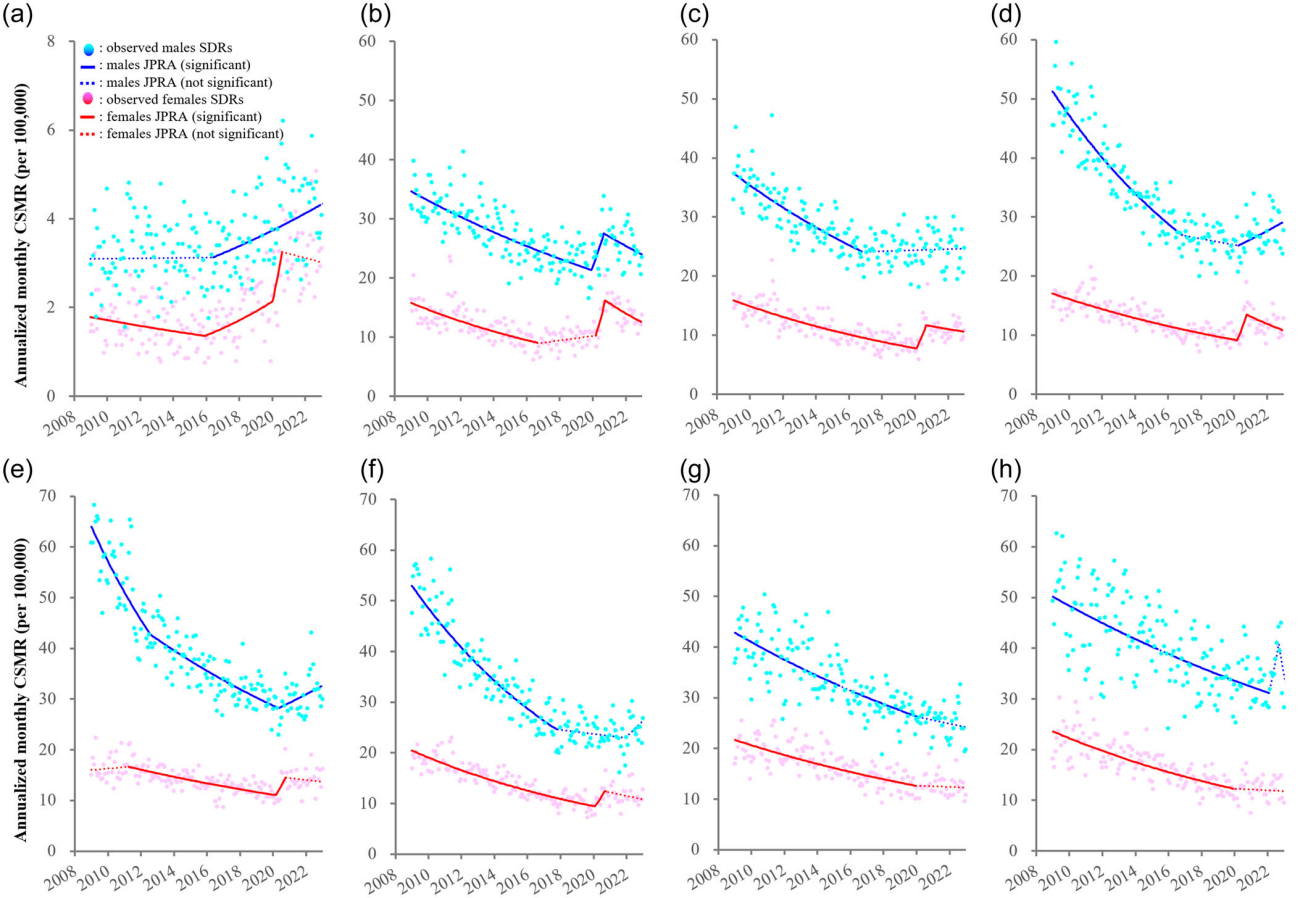
Fluctuations of annualized monthly CSMRs from January 2009 to June 2023 disaggregated by sex/age in Japan: (a) <20 years, (b) 20–29 years, (c) 30–39 years, (d) 40–49 years, (e) 50–59 years, (f) 60–69 years, (g) 70–79 years, and (h) >80 years. Blue and red circles annualized monthly CSMRs of males and females, respectively. Blue and red lines indicate the results calculated males and females CSMRs by JPRA, respectively. Solid and dotted lines indicate the significant and not significant trends of CSMRs detected by JPRA, respectively. CSMR, crude suicide mortality rate; JPRA, joinpoint regression analysis.

A number of Japanese studies have reported that males <30 years and females <70 years were at high risk for suicide during the pandemic.^12^, ^13^, ^15^, ^16^, ^17^, ^18^, ^19^, ^20^, ^21^, ^22^, ^23^, ^24^, ^25^, ^26^, ^27^, ^28^ Our previous studies using JPRA revealed that the temporal fluctuations in CSMRs were composed of various patterns (Figure 3):^12^, ^13^, ^14^, ^25^, ^26^, ^28^ (1) decreasing CSMR trends of males of 30–69 years of age were attenuated prior to the pandemic; (2) CSMR trends of males <20 years began to increase prior to the pandemic and continued increasing throughout the pandemic; (3) CSMR trends of females <30 years began increasing prior to the pandemic and sharply increased synchronously with the pandemic, followed by unchanging trends during the pandemic; (4) CSMR trends of males of 40–69 years of age increased throughout the pandemic; (5) decreasing CSMR trends of males of 70–79 years and females in their 70s and 80s were attenuated during the pandemic; (6) CSMRs of males of 20–29 years and females of 20–49 years sharply increased synchronously with the pandemic, followed by a decrease during the pandemic; and (7) CSMRs of males of 60–69 and >80 years increased in the late phase of the pandemic (Figure 3).

These findings suggest the existence of three types of suicidal vulnerability after the late 2010s. CSMRs of people under 20 years old began increasing before the pandemic, suggesting that risk factors unrelated to the pandemic were important for increasing suicides. CSMRs of males of 20–29 years and females <70 years sharply increased early in the pandemic but did not increase in the late stage. The CSMRs of males of 40–69 years probably increased during the late stage of the pandemic, therefore pandemic‐associated risk factors for suicide may include factors related to the pandemic and the end of the pandemic.

## CAUSALITY ANALYSES

### Basis of causality analyses for suicides

Recently, advances in computing power and software programs have made hierarchical linear models (HLMs, also known as multilevel models or split‐plot designs) the most popular statistical method for analyzing suicide causality. HLMs are appropriate for designs where data for subgroups are organized in nested data. Usually, the units (subgroups) of analysis are prefectures/municipalities that are nested within contextual/aggregate units (at the national level).^28^, ^34^, ^58^, ^59^, ^60^, ^61^ HLMs can provide alternatives to univariate or multivariate analyses of repeated measures. Occasionally, regression analyses yield results that are difficult to interpret, called “Simpson's paradox,”^62^ where significant temporal trends are observed for each group (fixed effects) but disappear/reverse when all groups are combined (random effects).^63^ Random effects are unique, time‐constant attributes of subgroups that are not correlated with the independent variables, whereas fixed effects are unique attributes of subgroups that do not vary over time. In particular, the HLM is considered to be a powerful application for determining fixed‐ and random‐effects.^64^

Vector autoregressive analysis (VAR) is powerful statistical model that can be used to detect temporal relations between multiple quantities that fluctuate over time.^65^ VAR generalizes the single‐variable autoregressive model by allowing for multivariate time‐series. Similar to AR models, each variable is composed of an equation that model changes over time. This equation consists of the lagged values of the variable, the lagged values of the other variables in the model, and an error term. VAR is simpler than structural models with simultaneous equations, since it only requires lists of variables that can be hypothesized to affect each other over time.^65^ VAR is usually summarized by structural analyses using Granger causality,^66^ impulse responses, and forecast error variance decompositions.^12^, ^13^, ^14^, ^35^ Granger causality is statistical hypothesis test for determining whether time‐series analysis is effective in forecasting the result.^66^ The impulse response explains the evolution of the model's variables in reaction to a shock in one or more variables.^67^ Forecast error variance decomposition—the extent to which the forecast error variance of each variable can be explained by exogenous shocks to the other variables in VAR—is used to aid in the interpretation of the VAR after fitting.^67^

HLMs and VAR are both powerful causality analysis tools; however, in addition to suicide statistics, they require other large independent variables in large databases (e.g., government databases). Suicide statistics collected by the National Police Agency (SSNPA) and basic data on suicide in the region (BDSR) published by the MHLW are government suicide databases that publish monthly suicide numbers within the next month, which is fast relative to other countries.^11^, ^68^ The SSNPA and BDSR are both considered to be as accurate and reliable suicide statistics databases. In Japan, judicial police must investigate the personal characteristics and background factors of each suicide case. The police investigate suicide motives based on evidence, suicide notes, official documentation (e.g., medical certificates and clinical recordings), and testimony from the victim's family. The results of their investigation discuss the different motives for suicide, which are compared with previously compiled lists of motives for suicide (52 subcategories in the SSNPA).^11^, ^14^, ^22^, ^25^, ^26^, ^68^ Time‐series analyses of temporal fluctuations of CSMRs disaggregated by suicidal motives in the SSNPA or BDSR can demonstrate temporal variation in causality, albeit indirectly through temporal fluctuation in suicidal motives.

### Impacts of unemployment

**Figure 4:**
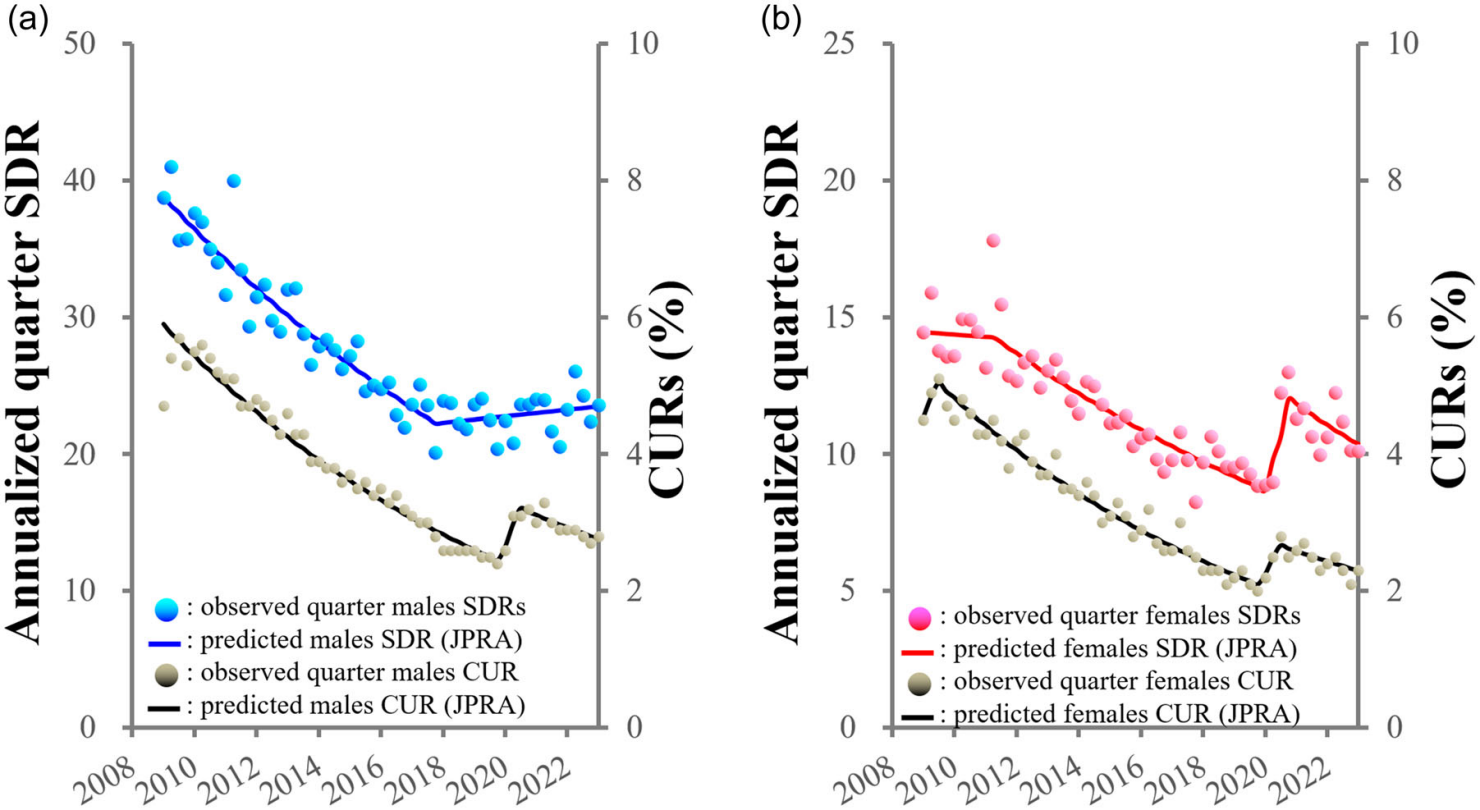
Fluctuations of annualized quarter SDRs and CURs of (a) males and (b) females in Japan from January 2009 to June 2023. Blue and red circles indicate the observed annualized quarter SDRs of males and females, respectively. Blue and red lines indicate the results calculated males and females SDRs by JPRA, respectively. Gray circles indicate the quarter CURs of males and females. Black lines indicate the results calculated males and females CURs by JPRA. CUR, completely unemployment rate; JPRA, joinpoint regression analysis; SDR, suicide death rate.

Unemployment is a well‐established risk factor for suicide and its causality has been analyzed.^43^, ^45^, ^69^, ^70^, ^71^, ^72^, ^73^, ^74^, ^75^, ^76^, ^77^ In Japan, completely unemployment rates (CURs) consistently linearly decreased from 5.5% to 2.5% in 2010–2019, whereas the CUR sharply increased synchronously with the pandemic to >3.0%, followed by a gradual decrease during the pandemic (Figure 4).^13^, ^51^ These temporal fluctuations of CURs seem to be similar to those of the female SDR/CSMR. Assuming that the sharply increasing female SDRs/CSMRs during the early stages of the pandemic in Japan were due to the increasing CUR, the rapidly increasing female SDRs/CSMRs in comparison to males during the early stage of the pandemic seem contrary to the fact that male suicides had been more sensitive to recession/unemployment than female suicides.^43^, ^71^, ^72^, ^73^, ^74^ Indeed, in Japan, male SDRs showed drastic increases from 26.6 (1997) to 37.1 (1998), while female SDRs increased from 12.4 (1997) to 15.3 (1998) at the Asian Financial Crisis.^2^, ^42^, ^43^, ^73^, ^74^ Therefore, the correlation of temporal fluctuations among SDR/CSMR and CUR cannot reasonably explain the discrepant fluctuations of male and female SDRs/CSMRs between the Asian Financial Crisis and the COVID‐19 pandemic.

In HLM analyses, before the pandemic, a random effect of CUR on CSMR could be detected in males in their 50s to 60s, but not in other groups of males or females.^8^ However, fixed‐effects HLM analysis detected interesting but unexpected relationships between CUR and SDR/CSMR before and during the pandemic. Before the pandemic (2009–2019), positive fixed effects of CUR on SDRs were detected in both males and females.^28^ During the pandemic (2020–2021), positive fixed effects of CUR on male SDR but not female SDR were observed.^28^ Therefore, despite the similar temporal fluctuations between CUR and female SDR, the discrepancies in the fixed effects of CUR on female SDR before and during the pandemic suggest that the similarity in temporal fluctuation patterns can suggest the possibility of causality, but cannot fully confirm definitive causality.

Before the pandemic, CURs disaggregated by unemployment duration (0–3, 3–6, 6–12, 12–24, and >36 months) linearly decreased. All CURs disaggregated by unemployment duration, other than 36 months, showed positive discontinuity (sharply increased) synchronized with the pandemic, while CURs decreased during the pandemic, falling until they were almost equal to prepandemic values in 2022.^13^ Therefore, the temporal fluctuation patterns of all CUR durations disaggregated by unemployment duration (with the exception of >36 months) showed a similar pattern to the female SDR/CSMR.^12^, ^13^, ^14^ VAR with impulse response analysis detected a positive temporal relationship between 6‐ and 12‐month periods of unemployment and the CSMRs of working‐age males (20–69), the positive impacts persisted for over 2 years. In contrast, the positive temporal relationship between an unemployment period of <6 months and the CSMRs of females of 20–49 years persisted for approximately 1 year.^13^ These findings can provide the temporal causality underlying the increasing CSMRs of working‐age females in the early pandemic period (from May to October 2020) and those of working‐age males in the late pandemic period (2020). Therefore, even a relatively shorter period of unemployment had a positive impact on females suicide, which persisted for approximately 1 year. However, suicide related to unemployment in males requires a longer period of unemployment, with the impacts persisting for >2 years.^12^, ^13^, ^14^, ^28^

In Japan, the duration for which unemployed individuals can receive unemployment benefits depends on the length of time that the individual has worked (i.e., individuals who have worked <1 year can receive benefits for 3 months and those who have worked >5 years can receive unemployment benefits for 1 year). Approximately 40% of part‐time employees have worked for <1 year.^78^ The part‐time employment rate in Japan is the highest (39.1%) among OECD countries (OECD average 25.3%).^79^ Part‐time employees are predominantly female in Japan,^78^ therefore part‐time female employees suffer from shorter cycles of employment and have shorter periods of unemployment benefit. The relationship between employment structures and unemployment benefit periods in Japan can plausibly indicate temporal causality from the increasing number of unemployed females who had worked for a shorter period of time in relation to the rapidly increasing CSMRs of females in from 20 to 49 years from the third to fourth quarters of 2020. A similar phenomenon of greater sensitivity of female suicides to economic recession in comparison to male suicides was observed in Hong Kong in 1997–2003 and is described as a “gender‐paradox.”^74^ Increasing women's labor force participation, including low‐skilled/low‐wage women (nonregular workers), is considered to be the underlying mechanism of the “gender paradox.”^73^, ^74^ Low‐skilled/low‐wage workers are usually the most expendable during an economic recession; consequently, women may be as affected as men (or more so) by deteriorating employment conditions.^73^, ^74^

The discrepancy between the Asian Financial Crisis (1997–1998) and the COVID‐19 pandemic (2020–2023) might be related to the transformation of the labor force participation structure in Japan.^13^ When labor force participation rates of females are low, the impacts of increasing CUR on females SDR/CSMR cannot be observed, whereas increasing females labor force participation has been reported to contribute to the positive association between CURs and CSMRs, resembling that of males.^80^ In Japan, the labor force participation rates of females during the Asian Financial Crisis (1997–1998) were approximately 60%, but they began increasing in the late 2010s and were >75% during the pandemic (those of males remained ~85% over time).^51^, ^81^ Taken together, the recent increase in female labor force participation may play an important role in the discrepancy between insensitivity of female CSMRs to increasing CURs during the Asian economic crisis and the converse high sensitivity observed during the early stage of the pandemic.^13^

### Candidate causes of the increasing CSMRs of adolescents/students

#### Temporal fluctuation of adolescent CSMRs caused by suicidal motives

Suicide is the fourth leading cause of death among 15–29‐year‐olds (adolescents) worldwide and is even more serious among youths in Japan, where it remained the leading cause of death among youths between 2009 and 2021.^31^, ^82^ Adolescence is a period of psychosocial and biological development that involves various social statuses (e.g., middle school, high school, university, and special vocational school students and working young adults).^83^ Although suicide among adolescents has been increasing over the long term, most studies from other OECD countries have reported that adolescent CSMRs decreased or remained unchanged during the pandemic.^84^, ^85^, ^86^, ^87^, ^88^ In contrast, in Japan, CSMRs of males and females of 10–19 years began to increase before the pandemic.^12^, ^25^, ^26^, ^28^ To date, there have been no causality analyses using independent variables that may affect the specific temporal fluctuations of adolescent CSMRs. However, temporal fluctuations in CSMR caused by suicidal motives may suggest, at least partially, factors underlying the increasing adolescent CSMRs.

It has been reported that schools, at least partially, contribute to suppressing adolescent CSMRs in Japan.^25^ CSMRs disaggregated by school age increased with age from middle school, high school, and university to special vocational school. The CSMRs of special vocational school students were almost equal to those of working‐age generations.^25^, ^26^ Notably, the CSMRs of male and female middle school students were almost equal, but the age‐dependent increase in CSMRs among male students was more pronounced than that among female students.^25^, ^26^ The primary causes of student CSMRs were worrying about the future and underachievement (in school‐related problems) and mental illness (depression and other mental illnesses).^25^, ^26^ School‐related problems were more impactable for CSMRs of male students than mental illness, whereas mental illness was more impactable for female students. Indeed, CSMRs caused by depression and other mental illnesses in middle‐school and high‐school females were greater than those in males, whereas the CSMRs caused by mental illness in males and females were almost equal in university and special vocational school students.^25^, ^26^ Considering that the onset of internalizing disorders occurs at approximately 15 years of age and the higher prevalence in female adolescents in comparison to males, internalizing symptoms/disorders probably play important roles in the greater CSMRs caused by these disorders in female students.^25^, ^26^, ^89^, ^90^

#### Impacts of GPSPP on student suicides

GPSPP and EFECBSC were reported to have contributed to the decrease in SDRs/CSMRs in 2009–2019 by enhancing regional welfare/social safety nets and improving regional vulnerabilities in social protection across communities, workplaces, and schools.^7^, ^8^, ^9^, ^10^, ^31^, ^91^ However, the CSMR of people under 20 years has not decreased.^12^, ^13^, ^14^, ^25^, ^26^, ^28^ The GPSPP establishes suicide prevention guidelines that are promoted by the government and have been revised approximately every 5 years (first: 2007–2012; second: 2012–2017; third: 2017–2022) according to the Plan‐Do‐Check‐Act cycle.^91^ To respond to adolescent suicide, the GPSPP has been revised to add the following priorities: “development of mental health support service in schools” in the first GPSPP (2007–2012) term, “enhancement of support and counseling systems for bullied children and victims of child abuse or sex crimes” in the second GPSPP (2012–2017), and “education on how high‐risk youth can request support” and “development of a suicide prevention program for children/adolescents” in the third GPSPP (2017–2022).^25^, ^91^ Therefore, the suicidal motives of adolescents, different from those of other generations of working‐age and the elderly, were insensitive to GPSPP.^25^, ^26^, ^92^

The impacts of the three GPSPP periods on student CSMRs disaggregated by suicidal motive from 2007 to 2022 was analyzed by ITSA with a dual interventions model (2012 and 2017).^25^ The CSMRs of high school and university students remained unchanged during the first GPSPP and decreased during the second GPSPP. However, they drastically increased during the third GPSPP. The analysis revealed similar fluctuation patterns of CSMRs caused by school‐related (underachievement) and health‐related (depression and other mental illness) motives in high school and university students of both sexes during the third GPSPP. CSMRs caused by conflict with parents consistently increased throughout the observation period in female high school and male university students. ITSA with a triple interventions model that added the pandemic in 2020 to the GPSPP periods detected additive impacts of the pandemic on increasing trends of some CSMRs during the third GPSPP, whereas it showed no independent impact on the increasing trends of student CSMRs during the third GPSPP.

The third GPSPP (2017–2022) listed new priority categories: “development of suicide prevention program for children/adolescents,” involving the enhancement of support systems for bullied/abused student/children in the school/community and “enlightenment of how to represent signs for seeking support against high‐risk student/children.”^91^ The 2022 White Paper on Suicide Prevention published by the MHLW reported that >25% of adolescents who suffered from depression or suicidal ideation were unable to seek nearby support by themselves.^31^ The third GPSPP captured future problems accurately. Although efforts to suppress CSMRs in adolescents/students have been made, the fact that these rates have not decreased suggests that the recent deterioration in psychosocial development or increasing internalizing disorder/symptoms in adolescents/students may play more significant roles in adolescent/student suicides than initially expected.^25^, ^26^ In addition to new additional priorities in the third GPSPP, enlightenment on how signs of seeking support are represented in high‐risk student/children, and enlightenment of schools, families and communities to the fact that students/children who are at risk due to internalizing symptoms/disorders cannot represent signs may also be important.

## OTHER POSSIBLE FACTORS

### Werther's effects and anomie

In the second half of 2020, female suicides by hanging at home increased regardless of region.^23^, ^24^ The MHLW repeatedly reminded various media sources to report on celebrity suicides in compliance with WHO guidelines^93^ (18 times during the pandemic).^94^ The MHWL estimated that increasing hanging suicides are probably imitative suicides.^95^ Increasing hanging suicides were observed from 1 month later and persisted for several months after celebrity suicides, indicating the characteristics of copycat suicides triggered by frequent reports of suicide of celebrities from mass media.^23^, ^24^, ^95^ Although the annual number of female suicides by hanging increased in 2020 (*n* = 47) compared with the average for 2015–2019 (*n* = 30.8 ± 2.4),^11^ the rise in suicides by hanging alone could not completely account for the increase in female suicides in 2020.^23^, ^24^

Interaction between imitative suicide (Werther's effect in a narrow sense)^96^ and anomie suicide (norm‐disruptive events) rooted in Durkheim's theory^97^ has been proposed as the main explanatory mechanism for Werther's effect,^95^, ^98^ and drawing conclusions about these complex relations remains a challenge.^99^ Durkheim hypothesized that in both economic recessions and booms, social systems are unable to sufficiently adapt to individual needs, leading to increasing suicides via weakened social integration.^80^, ^97^, ^98^ In fact, increasing CSMRs of females <60 years and males of 40–69 years of age coincided with the outbreak and the end of the pandemic, respectively.^12^, ^13^, ^14^ A study reported that the outbreak and end of the pandemic, including social restrictions, had heterogeneous psychological effects.^100^ Early in the pandemic, individuals suffered from stress due to forced drastic changes in lifestyle and social systems. Meanwhile, since late 2022, individuals have been forced to prepare to adapt to the “new normal” in the post‐COVID‐19 period due to the prospect of ending the pandemic.

The 2022 White Paper Information and Communications in Japan published by the Ministry of Internal Affairs and Communications reported that the delay in digitalization in Japan was a serious socioeconomic issue before the pandemic. However, during the pandemic, digitalization rapidly progressed out of necessity.^101^ Indeed, in 2022, the CSMRs of males (caused by failure at work) and females (caused by trouble adjusting to changing work environments) drastically increased.^4^, ^13^, ^14^, ^31^, ^68^ Although it is easy to understand that the aging population in Japan contributes to the lower degree of adaptation to changes in social structure (including rapid digitalization), further analyses should be conducted to investigate CSMRs caused by employment‐related motives since 2023.

### Decreasing birth rate with aging

The long‐lasting decrease in birth rates with aging is serious social problem in Japan that has led to the aging of the demographic structure with decreasing labor force, as well as an increasing number of only‐children.^101^, ^102^ It has been established that governmental financial expenditure on social welfare plays important roles in suicide mortality.^44^, ^59^, ^60^, ^103^, ^104^, ^105^, ^106^, ^107^, ^108^, ^109^, ^110^, ^111^ After the global financial crisis (2008), austerity policies implemented in several European countries led to increasing suicides via severe cuts in various regional welfare services.^44^, ^104^, ^105^, ^106^, ^107^, ^108^, ^109^, ^110^, ^111^ In Japan, government welfare and public health expenditure was effective for controlling suicide mortality before the pandemic, but welfare was more impactable, as demonstrated using HLMs.^59^, ^60^, ^61^ In the welfare subdivisions, child welfare and social welfare were effective in decreasing suicides, but elderly welfare unexpectedly contributed to increasing suicides.^59^, ^60^, ^61^ It can be speculated that governmental expenditure shifted to public health during the pandemic. The publication of reports on national and regional governmental expenditure from 2020 to 2023 is awaited to clarify the relationships in detail.

Although it is outside the scope of this review, a brief discussion regarding the decreasing birth rates as remaining challenges for the future should be included. Statistical analyses indicated that internalizing symptoms/disorders play important roles in increasing adolescent/student suicides.^25^, ^26^ Long‐lasting, progressively decreasing birth rates have continuously increased for only‐children without siblings, with rates of >20% in 1997 and >30% in 2015.^102^, ^112^ Historically, the emergence of second children/siblings had been argued to contribute to developmental crisis for firstborn children by earlier psychodynamics^113^, ^114^; however, while the birth of a second child/sibling probably provides challenges to very young firstborn children it may also lead to rapid development. Consequently, not all children experience a crisis.^115^, ^116^, ^117^, ^118^ Firstborn children with siblings display positive or regular responses with quick adaptation to new tasks, more positive emotions, and less separation reaction and dependent behaviors in comparison to only‐children.^119^, ^120^, ^121^ Therefore, children raised with siblings may be less affected by the stressful environment and/or gain flexibility.^119^ A recent Chinese study reported that scores associated with internalizing, externalizing, emotional, and behavioral problems of only‐children were higher than those of firstborn children with siblings.^120^

It is easy to understand that parents tend to adopt strategies of high‐care/high‐control in relation to an only‐child.^25^, ^26^, ^113^, ^114^ Parker categorized parenting attitudes into two dimensions, “care” (consideration, concern, and affection expressed by the parent) and “control” (intrusiveness, protection, and manipulation unrelated to affectionate feelings).^122^ Low‐care parenting has traditionally been considered to negatively impact developmental models regarding the competent/worthy self‐model and reliable/supportive model of others with persistence to adolescence, resulting in increasing risks of internalizing disorders and suicide.^122^, ^123^, ^124^, ^125^, ^126^, ^127^, ^128^, ^129^, ^130^, ^131^ The depressive mood and hopelessness of university students are positively related to “high‐control” and “low‐care.”^132^ Furthermore, mothers with internalizing symptoms/disorders tend to have attitudes with affectionless control styles (low‐care/high‐control).^132^ These findings suggest the possibility that maternal internalizing symptoms/disorders may induce a vicious negative cycle that increases the prevalence of internalizing disorders in later generations.^25^ Indeed, the patient survey published by the MHLW reported an increasing prevalence of internalizing disorders in individuals of 10–24 years in 2020 compared with 2017.^25^, ^26^, ^133^

## CONCLUSION

Based on suicide statistics in Japan from January 2009 to June 2023, this review discusses the mechanisms underlying decreasing suicides in 2009–2019 and increasing suicides in 2020–2023. Political authorities in Japan rapidly implemented enhancements of unemployment measures, support for small‐to‐medium‐sized enterprises, and mental/social support programs to respond to psychosocial/socioeconomic deterioration by leveraging existing comprehensive suicide prevention programs, according to concerns of economics, psychiatry, and public health. Despite these efforts, suicides increased for 3 years during the pandemic. This review suggests that traditional established suicide risks alone cannot fully explain the increasing number of suicides in Japan since 2020. In particular, the recent increase in the social participation rate of females has played an important role in the increasing number of suicides among working‐age females via the enhanced sensitivity of female suicides to short‐term unemployment during the initial stage of the pandemic. In contrast, male suicides, which were relatively stable during the initial stage of the pandemic, increased in the second half of 2022. It cannot be denied that in addition to long‐term unemployment, the drastic changes in the workplace during the pandemic, including digitalization, may have contributed to this increase. Most importantly, increasing internalizing symptoms/disorders in adolescents/students due to decreasing birth rates/increasing only‐children or parenting attitudes (high control) has played an important role in increasing adolescent/student suicides since the late 2010s.

## AUTHOR CONTRIBUTIONS

Motohiro Okada had full access to all of the data in the study and takes responsibility for the integrity of the data and the accuracy of the data analysis. Motohiro Okada: concept and design, acquisition, analysis, and interpretation of data, drafting of the manuscript, critical review of the manuscript for important intellectual content, statistical analysis, obtained funding, administrative, technical, or material support, and supervision. Ryusuke Matsumoto: concept and design, acquisition, analysis, and interpretation of data, drafting of the manuscript, critical review of the manuscript for important intellectual content, statistical analysis, and obtaining funding. Eishi Motomura: concept and design, acquisition, analysis, and interpretation of data, drafting of the manuscript, critical review of the manuscript for important intellectual content, statistical analysis, obtained funding, administrative, technical, and material support, and supervision.

## CONFLICT OF INTEREST STATEMENT

The authors declare no conflicts of interest.

## ETHICS APPROVAL STATEMENT

The medical ethics review committee of Mie University waived the requirements for informed consent and ethical approval because the study used data that are available from publicly accessible governmental databases.

## PATIENT CONSENT STATEMENT

N/A

## CLINICAL TRIAL REGISTRATION

N/A

## Data Availability

All raw data are publicly available to any persons via Japanese national databases for the Basic data on suicide in the region (https://www.mhlw.go.jp/stf/seisakunitsuite/bunya/0000140901.html) and the Labor Force Survey (https://www.stat.go.jp/data/roudou/sokuhou/tsuki/index.html) published by MHLW.
